# Insights into the mechanism of growth and fat deposition by feeding different levels of lipid provided by transcriptome analysis of swamp eel (*Monopterus albus*, Zuiew 1793) liver

**DOI:** 10.3389/fimmu.2023.1118198

**Published:** 2023-06-19

**Authors:** Yazhou Zhang, Feng Guo, Xin Yang, Yu Liu, Yihong Bao, Zirui Wang, Zhonghua Hu, Qiubai Zhou

**Affiliations:** ^1^ College of Animal Science and Technology, Jiangxi Agricultural University, Nanchang, China; ^2^ Key Laboratory of Featured Hydrobios Nutritional Physiology and Healthy Breeding, Nanchang, China; ^3^ School of Economics and Management, Jiangxi Agricultural University, Nanchang, China

**Keywords:** fat deposition, growth performance, lipid level, *Monopterus albus*, transcriptomics

## Abstract

Lipid is an important source of energy in fish feeds, and the appropriate fat content can improve the efficiency of protein utilization. However, excessive lipid content in the feed can lead to abnormal fat deposition in fish, which has a negative effect on the growth of fish. Therefore, the effects of feed lipid levels on swamp eel were studied. Essential functional genes were screened using transcriptomics. We divided 840 fish into seven groups (four replicates). A mixture of fish and soybean oils (1:4), 0%, 2%, 4%, 6%, 8%, 10%, and 12% was added to the basic feed were named groups one to seven (L1-L7), respectively. Isonitrogenous diets were fed swamp eel for 10 weeks. Growth performance, visceral index, nutritional components, and biochemical indexes were measured and analyzed. Livers of the 0%, 6%, and 12% groups were subjected to transcriptome sequencing analysis. The results of our study showed that: the suitable lipid level for the growth of swamp eel was 7.03%; the crude fat content of whole fish, liver, intestine, muscle, and skin increased with the increase of lipid level, with some significant difference, and excess fat was deposited in skin tissue; triglyceride, total cholesterol, and free fatty acid contents increased with the increase of feed lipid level. High-density lipoprotein levels in the L3 and L4 groups were higher than in the other groups. Blood glucose concentrations in the L5, L6, and L7 groups increased; the liver tissue structure was damaged when the lipid level was too high. two-hundred-and-twenty-eight differentially expressed genes were found. Several critical pathways regulating glucose metabolism and energy balance (e.g., glycerolipid metabolism, glycolysis synthesis, degradation of ketone bodies, and Janus Kinase/Signal Transducer and Activator of Transcription signaling pathway) were enriched in swamp eel compared with the Kyoto Encyclopedia of Genes and Genomes (KEGG) database. Suitable lipid levels (7.03%) can promote the growth of swamp eel, and excessive lipid levels can cause elevated blood lipids and lead to liver cell damage. Regulatory mechanisms may involve multiple metabolic pathways for glucose and lipid metabolism in eels. This study provides new insights to explain the mechanism of fat deposition due to high levels of lipid and provides a basis for the production of efficient and environmentally friendly feed for swamp eel.

## 1 Introduction

Lipids are essential macronutrients that regulate animal growth, reproduction, health, and body functions (1). As an efficient energy source, lipids provide optimal growth rates for fish (2). Optimal levels of dietary lipids can have the effect of sparing protein, which can maximize nitrogen retention and benefit growth (3, 4). In recent years, in order to save protein sources and improve economic benefits, high-fat diet has been widely used in aquaculture (5–7). However, high-lipid diets can lead to metabolic disturbances, fat accumulation, inflammation, reduce digestive function and feed utilization, retarding fish growth and development (8–11).

Previous studies have shown that a high-fat diet can alter the expression of genes associated with lipid metabolism in the liver, leading to fat deposits in the liver (12, 13).

Fatty liver injury caused by excessive fat deposition is one of the common liver diseases in cultured fish in the world, especially in China, which can lead to metabolic dysfunction, poor disease resistance and even death of fish (14). In recent years, liver damage caused by high fat diet has attracted the attention of many researchers. Lipid metabolism is a complex biochemical pathway involving many vital enzymes and transcription factors, including lipid digestion, absorption, transport, synthesis, and decomposition (15, 16). In recent years, a new generation of high-throughput sequencing technology (RNA-seq sequencing technology) has been increasingly used to study these complex physiological processes (17).

Swamp eel (*Monopterus albus*, Zuiew 1793) is distributed principally in eastern and southern Asia, the southeastern United States, and northern Australia (18). This fish is an essential economic species and one of the major aquaculture species in Asia because of its high nutritional value and potential medicinal value (19). As the wild resources of swamp eel are declining, farmed swamp eel has taken up an increasing proportion in China. According to the China Fishery Yearbook, during the 16 years from 2007 to 2022, the yield of swamp eel farming in China increased from 196,190 tons to 311,000 tons (the highest yield was 386,137 tons). In their natural state, individuals of this species are carnivores that feed on swamps and small aquatic animals (20). Wild garbage fish and pellet feed are used to cultivate swamp eels. The production of artificial feed usually uses substantial amounts of fishmeal and oil. The nutritional imbalance affects the growth and reproduction performance of swamp eel, but the specific cause is unknown. It has been suggested in numerous reports that lipids play an important role in fish growth and reproduction (21). Too high or too low lipid levels can lead to fatty liver disease, or gonadal development is not synchronized with gender reversal. However, less knowledge was available about the requirements and utilization of lipid levels during swamp eel growth. This is significantly detrimental to the development of the swamp eel aquaculture industry. Therefore, clarifying the optimal lipid level required by swamp eel and exploring the mechanism of fat deposition due to high fat diet are beneficial to the development of efficient feeds for swamp eel.

## 2 Materials and methods

### 2.1 Diet preparation

Fish meal, puffed soybean meal, and compound protein were used as intact protein sources; wheat and puffed corn were used as the energy feeds; after the fish oil and soybean oil are mixed in proportion, they are added to the feed in a gradient of 0%, 2%, 4%, 6%, 8%, 10% and 12% to prepare 7 kinds of isonitrogenous feeds, which are respectively recorded as L1, L2, L3, L4, L5, L6, and L7. The feed ingredients were ground into a fine powder and sieved through 80-mesh size, fully mixed and processed into 2 mm pellets with a puffing feed machine, dried naturally, and stored in a refrigerator at -20°C for use. The experimental feed formula and main nutrients are shown in **Table 1**, in which the crude lipid contents of each group were 2.96% (L1), 5.28% (L2), 6.64% (L3), 8.23% (L4), 10.51% (L5), 13.37% (L6) and 15.40% (L7), respectively.

**Table 1:** Feed formulation and nutrient levels of trial diets(%).

Ingredients	Lipid level						
	L1	L2	L3	L4	L5	L6	L7
Fish meal	27.2	27.2	27.2	27.2	27.2	27.2	27.2
Compound protein	22	22	22	22	22	22	22
Puffed soybean meal	18	18	18	18	18	18	18
Casein	3	3	3	3	3	3	3
Puffed corn	6	6	6	6	6	6	6
Calcium dihydrogen phosphate	2	2	2	2	2	2	2
*Premixture	1.5	1.5	1.5	1.5	1.5	1.5	1.5
Choline	0.3	0.3	0.3	0.3	0.3	0.3	0.3
Wheat	12	11	10	9	8	7	6
Microcrystalline cellulose	8	7	6	5	4	3	2
Oil	0	2	4	6	8	10	12
**Nutrition							
Crude protein	41.02	40.89	40.87	41.05	40.75	40.81	40.79
Crude lipid	2.96	5.28	6.64	8.23	10.51	13.37	15.40
Crude ash	13.01	13.15	13.59	13.37	13.28	12.99	13.61

*Premix reference NRC (1993); **Nutritional components measured.

### 2.2 Feeding trial and experimental conditions

Healthy juvenile swamp eels were obtained from the College Of Animal Science And Technology Of JiangXi Agricultural University in China. Fish weighed 22.00-25.00 g were brought to the laboratory and acclimatized to the conditions for 10 days. De-chlorinated aerated tap water was adopted and maintained at 28 ± 2°C, dissolved oxygen above 4 mg/L, and ammonia nitrogen below 0.02mg/L. The photoperiod was in a 14 h light/10 h dark cycle. Before the experiment, the fish were fasted for 24 h and grouped after anesthesia by eugenol (Shanghai, China). 20 fish of the same size were randomly distributed into a fiberglass tank (80cm×60cm×50cm). Each diet was randomly assigned to four replicate groups of fish. The daily diet was fed by hand at 2-3% of body weight at 18:00 every day. The survival rate of eels in all groups was above 95% during the whole culture period, and there was no significant difference among all groups. All experimental procedures were carried out in accordance to the guidelines in the China Law for Animal Health Protection and Instructions (Ethics approval No. SCXK (YU2005-0001)).

### 2.3 Tissue sampling

Fasting 24 h after the end of the experiment, the total weight of the fish in each cylinder was weighed, then the final body weight (FBW), weight gain rate (WGR), specific growth rate (SGR), and feed conversion ratio (FCR) were calculated. Four fish were taken to take blood, the weight of viscera, liver, intestine, and spleen was weighed, and the body weight and body length were measured. Then, the condition factor (CF), viscerasomatic index (VSI), hepatosomatic index (HSI), enteric index (EI), splenic index (SI), and protein efficiency (PER) were calculated. Six additional fish/group (two fish/tank) were chosen for the collection of liver tissue samples for gene expression analysis. These samples were immediately frozen in liquid nitrogen and stored at −80°C for further gene expression analysis.

### 2.4 Chemical composition and biochemical analysis

Moisture and crude lipid in basal diet were measured according to AOAC, 1990 standard methods. Commercial kits (Leadman Co., Ltd) for triglyceride (TG), total cholesterol (TC), free fatty acid (FFA), high-density lipoprotein (HDL), low-density lipoprotein (HDL) and glucose (GLU) were used to detect serum parameters by Beckman AU480 biochemistry analyzer. The activities of glutathione peroxidase (GPX) and glutamic oxalacetic transaminase (GOT) were measured by the commercial kit (Nanjing Jiancheng Biotechnic Institute, China) according to the manufacturer’s instructions.

### 2.5 Liver histopathology

The liver samples fixed with 10% formalin for 12-24 h, dehydrated in alcohol series (70%-100%) and infiltrated in dimethyl benzene, and embedded in paraffin. 0.5 μm thick tissue sections were obtained with a microtome. Stained with haematoxylin-eosin and examined and documented using a computerized image analyzer(Olympus, Japan). A computerized image analyzer (Leica LAS Interactive Measurements) was used to evaluate the hepatocyte morphology and to measure the average area of these cells. Images were acquired using LAS software to assess the morphology of hepatocytes and to measure the average area of these cells. 15 cells were quantified per section and 3 sections were used per sample(n = 3 animals per tank; n = 45 cells per animal measured). The LAS software was also used to obtain the measurements of the average hepatocyte area. Similar analyses have been performed in other teleost species with different tissues (22, 23).

### 2.6 RNA extraction, library construction, and sequencing

The total RNAs from liver tissue were extracted using Trizol reagent (Invitrogen, U.S.) according to the manufacturer’s protocol. The quality and quantity of RNA were measured by spectrophotometer and agarose gel electrophoresis (0.8% w/v). Then, the library was constructed using the Illumina Seq RNA Sample Preparation Kit (Illumina, USA). Nine cDNA libraries were submitted for Illumina HiSeqTM2500 sequencing analysis following the manufacturer’s instructions.

### 2.7 Data of RNA-seq processing and sequence annotation

Invalid readings in sequencing data were discarded to avoid a negative impact on subsequent bioinformatics analysis using fast QC. The remaining clean labels were mapped to the *Ct. Idella* reference genome (http://www.ncgr.ac.cn/swampeels) using HISAT (version 0.1.6) (24). The HTSeq calculated the number of aligned reads per exon through annotation of the *Ct. idella* genome.

### 2.8 Identification of differentially expressed genes

Differentially expressed genes were analyzed in CLC Genomics Workbench and high-quality readings from nine samples were mapped to the Trinity reference component. The total mapped reads for each transcript were determined and then normalized to detect the Reads Per Kilobase exon model per Million (RPKM) of mapped reads. After scale normalization of RPKM values, calculate fold changes in different expressions (25).

### 2.9 Gene ontology analysis of DEGs

Screening overrepresented GO annotation Ontologizer 2.0 in differentially expressed genes using GO analysis and enrichment analysis (26). GO annotation was obtained using the GOseq and WEGO for each differentially expressed gene (27). GO enrichment analysis was performed using singular enrichment analysis (SEA) by comparing DEGs to all expressed genes. The threshold was set as False discovery rate (FDR) < 0.001. Kyoto Encyclopedia of Genes and Genomes (KEGG) pathways including differentially expressed genes (DEGs) were identified based on GO analysis (28). After multiple trials, it was considered that the pathway with Q-value < 0.05 was significantly enriched in DEGs. Bioinformatic analysis was performed using Omicsmart, a dynamic and interactive online platform for data analysis (https://www.omicsmart.com).

### 2.10 Validation of quantitative real-time PCR

To validate RNA-seq data, RNA samples for the transcriptome were detected using quantitative real-time PCR (qRT-PCR) assays by SYBR Premix Ex Taq (TOYOBO, Japan). All experiment procedures were performed according the standard procedures established in our lab (29). In brief, total RNA was extracted from tissues using Trizol (Omega, US) and digested with DNase I (New England, USA). cDNA was generated by M-MLV Reverse Transcriptase (Thermo Fisher Scientific, US). The mRNA expression levels were determined with qRT-PCR using an SYBR Premix Ex Taq (TOYOBO, Japan). The qRT-PCR amplifications were carried out in a 20 μL solution including 1 μL cDNA template, 10μL SYBR Premix Ex Taq, 0.6μL forward and reverse primer, and 7.8 μL ddH_2_O. All samples were run in duplicate and carried out with the ABI 7500 Real-Time PCR System using the program: 95°C for 30 s, 40 cycles of 95°C for 10 s, 56°C for 34 s, and 72°C for 30 s. The housekeeping genes, beta-actin, were used as the internal control, and its expression remained stable throughout the study. The relative gene expression levels were calculated using the 2^-△△Ct^ method and compared with beta-actin gene levels (30). The primers used in this study are shown in **Supplementary Table 1**.

### 2.11 Statistical analysis

The following variables were calculated:

Weight gain rate (WGR, %) = (Wt-Wo)/Wo × 100.

Specific growth rate (SGR, %d) = (LnWt – LnWo) × 100/t.

Feed conversion ratio (FCR) = feed intake/(Wt-Wo).

Protein efficiency ratio (PER, %) = (Wt-Wo)/(feed intake × feed protein content)

Hepatosomatic index (HSI, %) = (liver weight/Wt) × 100.

Viserosomatic index (VSI, %) = (visceral weight/Wt) × 100.

Enteric index (EI, %) = (Intestine weight/Wt) × 100

Splenic index (SI, %) = (Spleen weight/Wt) × 100.

Condition factor (CF, %) = 100 × (Wt-Wo)/(body length)^3^.

Where Wt and Wo were final and initial fish weight, respectively; t was duration of experimental days.

All data were presented as the mean ± SEM. Statistical analysis was performed with SPSS19.0 software. On the basis of one-way analysis of variance (ANOVA), Duncan’s method was used for multiple comparisons, and the significance level was *P*<0.05. Regression analysis is used to simulate the relationship between fish weight gain, feed coefficient and feed lipid level.

## 3 Results

### 3.1 Effects of different lipid levels on the growth performance of swamp eels

**Figure 1:**
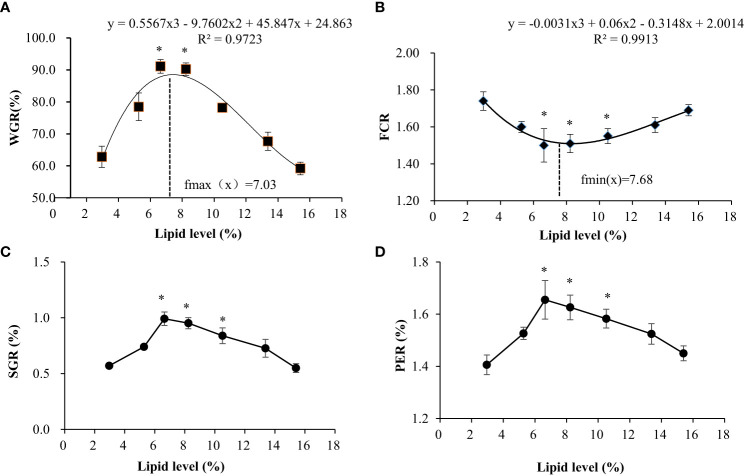
Effects of dietary lipid level on growth performance of *Monopterus albus.* **(A)** Comparison of weight gain rate among test groups; **(B)** Comparison of feed coefficient among test groups; **(C)** Comparison of specific growth rates among test groups; **(D)** Comparisons of protein efficiency among test groups. * Indicates the groups with the best feeding results under the corresponding index.

As shown in **Figure 1**, different feed lipid levels significantly affect the WGR, SGR, and PER of swamp eels. With the increase in lipid content in the feed, these indices had a trend of increasing and then decreasing, reaching the maximum in the L3 and L4 groups. Precisely, the opposite occurred with feed FCR. Regression simulation analyzes the relationship between the weight gain rate and feed lipid level of the swamp eel. It was shown in these results that when the feed lipid level was 7.03%, the weight gain rate was the largest. Therefore, under experimental conditions, the appropriate feed lipid level for swamp eel is 7.03%.

### 3.2 The effect of different lipid levels on the physical indicators of swamp eels

Further research results are shown in **Table 2**. The change in dietary lipid content had an insignificant effect on the physical indicators of swamp eel. The changing trend of the VSI and HSI of each group was the same, but the L1 group was significantly smaller than the other six groups (*P* < 0.05), and there was no significant difference among groups L2-L7. Additionally, the L4 group had the largest EI, and the L7 group had the largest SI, which was significantly higher than the other six groups (*P* < 0.05). There was no significant change in the CF of each group.

**Table 2:** Effect of dietary lipid level on figure and visceral index of *Monopterus albus*(n=12).

Groups	Wt	VSI(%)	HIS(%)	EI(%)	SI(%)	CF(g/cm^3^)
L1	27.38 ± 0.53^ab^	4.51 ± 0.25^a^	2.34 ± 0.35^a^	1.96 ± 0.07^a^	0.29 ± 0.02^a^	0.10 ± 0.01
L2	30.05 ± 0.69^c^	6.77 ± 0.20^b^	3.82 ± 051^b^	2.16 ± 0.18^a^	0.29 ± 0.03^a^	0.09 ± 0.02
L3	32.14 ± 0.34^d^	6.85 ± 0.15^b^	4.44 ± 0.11^b^	2.20 ± 0.15^a^	0.29 ± 0.02^a^	0.09 ± 0.01
L4	31.98 ± 0.34^d^	7.12 ± 0.46^b^	4.67 ± 0.89^b^	2.52 ± 0.07^b^	0.32 ± 0.04^a^	0.10 ± 0.02
L5	29.96 ± 0.16^c^	6.28 ± 0.73^b^	4.28 ± 0.16^b^	2.09 ± 0.11^a^	0.31 ± 0.03^a^	0.09 ± 0.01
L6	28.16 ± 0.47^b^	5.70 ± 0.23^b^	4.37 ± 0.05^b^	2.27 ± 0.22^a^	0.31 ± 0.03^a^	0.09 ± 0.02
L7	26.76 ± 0.57^a^	5.12 ± 0.60^b^	4.44 ± 0.45^b^	2.15 ± 0.10^a^	0.38 ± 0.04^b^	0.10 ± 0.02

Values are mean± S.E. different letters means significant difference (P<0.05).

### 3.3 The effect of different lipid levels on the nutrient content of swamp eels

**Figure 2:**
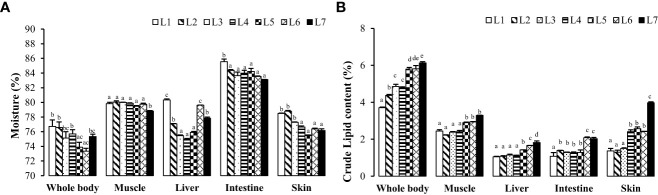
Effects of dietary lipid level on nutritional components of tissues of *Monopterus albus.* **(A)** Effects of feed lipid level on moisture content in different tissues; **(B)** Effects of feed lipid level on crude lipid content in different tissues. Values are mean± S.E. different letters means significant difference (P<0.05).

As seen in **Figure 2**, with the increase in fat levels, the water content of the whole fish, liver, intestine, muscle, and skin of each group showed a downward trend, and the crude fat content showed an upward trend. The crude fat content of whole fish, muscle, and liver in the L6 and L7 groups was significantly higher than that of the other groups (*P* < 0.05). The crude fat content of the skin increased the most, ranging from 1.37% to 3.97%, with an increase in fat levels.

**Figure 3:**
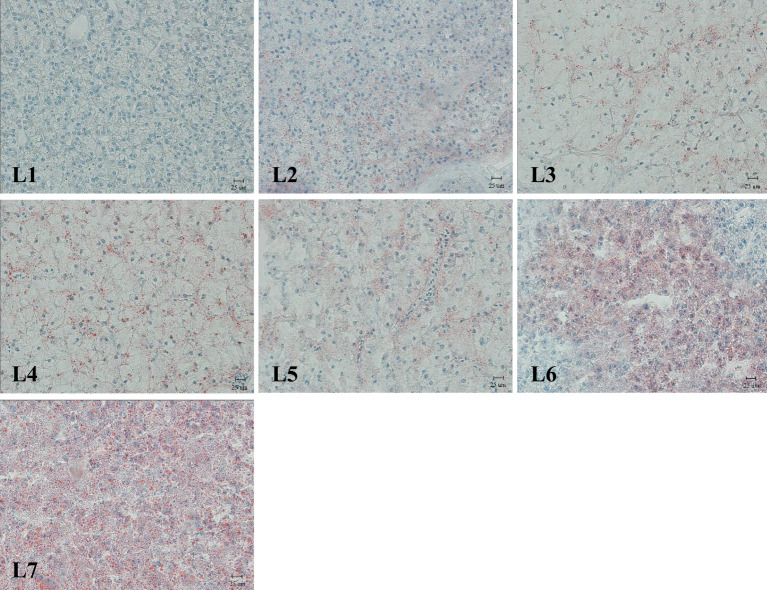
Liver lipid deposition, Oil red O dyeing, Magnification**×**400.(Lipid is dyed red).

The liver was stained with Oil Red O (**Figure 3**). Fat deposition in the liver tended to increase to observe the liver fat deposition of rice field eel in each experimental group. The liver fat deposition in the L1 and L2 groups was low, and the L3–L5 groups were stained as the fat of the red particles began to increase and was dispersed. The two groups of L6 and L7 were the largest and appeared in the flakes.

### 3.4 Effects of different lipid levels on serum biochemical parameters of swamp eels

The effects of different lipid levels in the feed on the serum biochemical parameters of swamp eels are shown in **Table 3**. The enzyme activities of GPT and GOT in the serum of the L6 and L7 groups were significantly greater than those of the other five groups (*P* < 0.05). The concentration of TG and TC in serum increased with the increase in feed fat level, and L6 and L7 were significantly higher than in the other five groups (*P* < 0.05). The three groups of L5, L6, and L7 had the largest FFA concentration, which was significantly higher than the other four groups (*P* < 0.05). The content of HDL in serum showed a trend of rising first and then falling, with the highest range in the L3 and L4 groups, and the LDL content of the L1 group was significantly lower than that of the other groups (*P* < 0.05). The blood glucose concentration of the L5–L7 group was significantly higher than that of group four (*P* < 0.05).

**Table 3:** Effect of dietary lipid level on serum biochemical indexes of *Monopterus albus.*.

Index	Groups						
	L1	L2	L3	L4	L5	L6	L7
GPT (IU/L)	0.24 ± 0.02^a^	0.27 ± 0.02^a^	0.21 ± 0.01^a^	0.24 ± 0.03^a^	0.28 ± 0.02^a^	0.31 ± 0.01^b^	0.55 ± 0.03^c^
GOT (IU/L)	9.89 ± 1.46^a^	13.26 ± 1.05^a^	12.41 ± 0.45^a^	12.69 ± 1.08^a^	21.44 ± 0.48^b^	22.44 ± 2.31^b^	23.41 ± 1.10^b^
TG (mmol/L)	0.24 ± 0.01^a^	0.27 ± 0.02^a^	0.45 ± 0.02^b^	0.42 ± 0.04^b^	0.41 ± 0.02^b^	0.79 ± 0.02^c^	0.98 ± 0.06^d^
TC (mmol/L)	3.82 ± 0.03^a^	4.33 ± 0.25^b^	4.82 ± 0.02^c^	4.87 ± 0.05^c^	4.84 ± 0.17^c^	5.37 ± 0.07^d^	6.24 ± 0.22^e^
NEFA (μmol/L)	27.57 ± 1.25^a^	30.08 ± 4.34^ab^	35.09 ± 2.51^ab^	40.10 ± 3.32^bc^	47.62 ± 3.32^cd^	50.13 ± 4.52^cd^	55.12 ± 3.32^d^
HDL-C (mmol/L)	1.23 ± 0.08^b^	1.49 ± 0.04^c^	2.02 ± 0.14^d^	1.81 ± 0.08^d^	0.96 ± 0.09^a^	0.97 ± 0.03^a^	0.84 ± 0.04^a^
LDL-C (mmol/L)	1.17 ± 0.08^a^	1.52 ± 0.07^b^	1.64 ± 0.08^b^	1.64 ± 0.02^b^	1.44 ± 0.06^b^	1.60 ± 0.04^b^	1.58 ± 0.05^b^
GLU (mmol/L)	1.94 ± 0.01^b^	1.73 ± 0.02^a^	1.75 ± 0.01^a^	1.75 ± 0.01^a^	2.37 ± 0.01^c^	2.39 ± 0.01^c^	2.55 ± 0.01^d^

Values are mean± S.E. different letters means significant difference (P<0.05).

### 3.5 The effect of different lipid levels on the liver tissue structure of swamp eels

**Figure 4:**
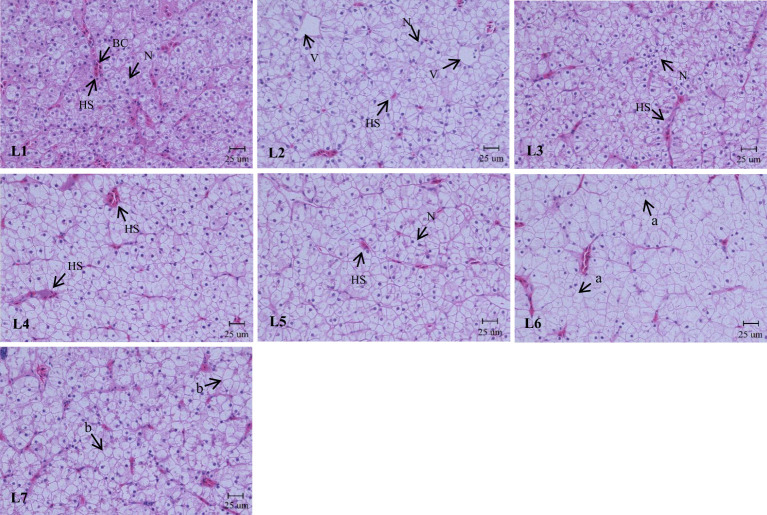
Effects of dietary lipid level on liver microstructure of *Monopterus albus*, HE staining. Magnification**×**400. V: central vein HS: hepatic sinusoid BC: blood corpuscle N: nucleus. a: nucleus disappearance b: lipid vacuoles.

As shown in **Figure 4**, the liver structure in the L1–L4 groups was complete, and the lobular structure was prominent. The liver cells are arranged radially around the central vein. Liver cells were tightly arranged, cell boundaries were clear, and most nuclei were in the center of the cell. The nuclei of the cells in the L5 group began to decrease gradually, and the phenomenon of nuclear deviation appeared. In the L7 group, the arrangement of hepatocytes was disordered, the hepatocyte cord was not obvious, the nucleus was on one side, and white transparent vacuoles appeared.

**Figure 5:**
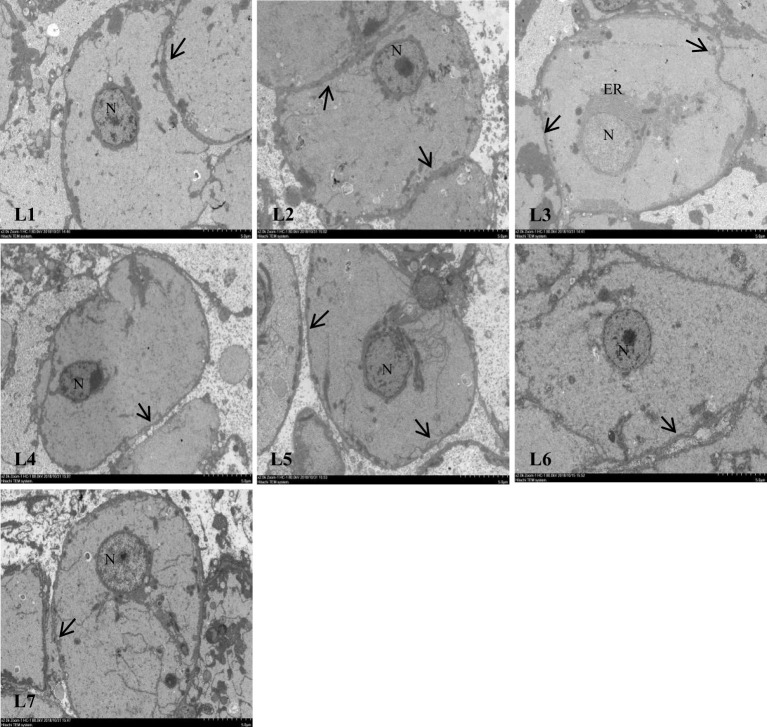
Effect of feed lipid level on liver cell junction space of *Monopterus albus*. N: nucleus ER: endoplasmic reticulum;The arrow shows the junction of hepatocytes.

Different lipid levels in the feed also impacted cellular structure of swamp eels (**Figure 5**). The connections between the liver cells in the L1–L3 group were tight. Still, as the lipid level continued to increase, the relationships between the liver cells became discrete, and the gap widened.

### 3.6 Transcriptomics sequence evaluation

To further reveal the molecular mechanism of the effects of different lipid levels on the metabolic pathways of swamp eel, we performed liver transcriptome sequencing analysis of the 0% (L1), 6% (L4), and 12% (L7) groups. Sequencing of the liver transcriptome of swamp eel fed with three groups of different lipid levels obtained raw data of 18674437800 bp. After removing the low-quality, short sequence and linker sequence in the original sequence, 39081412, 41585768, and 43829972 clean reads were obtained respectively. Among them, the proportions of reads in the LF, MF, and HF group data corresponding to the swamp eel genome were 87.31%, 79.44%, and 84.73% respectively, of which the only ones corresponded to 83.68%, 76.13%, and 81.85% respectively.

Sequencing saturation analysis can reflect a certain extent whether the amount of sequencing data meets the demand. When the amount of sequencing reaches a certain level, the increase in the number of detected genes gradually slows down, indicating that the number of detected genes tends to be saturated (**Supplementary Figure 1**). The saturation results of the samples in the L1, L4, and L7 groups are similar, and the genes compared by Clean Reads no longer increase, indicating that the number of sequencing sequences tends to be saturated.

Gene coverage refers to the proportion of genes covered by reads, and its value is equal to the ratio of the number of bases covered by unique mapping reads in each gene to the bases contained in the gene. Our results (**Supplementary Figure 2**) show that the predicted genes with a coverage of 80% to 100% in the L1, L4, and L7 groups are 23463, 22561, and 20362, respectively; and the coverage of 60% to 80% is 5016, 5269, and 5481, respectively; Coverage between 40% and 60% is 2729, 2755, and 2989; coverage between 20% and 40% is 2241, 2374, and 2778; coverage less than 20% is 2699, 2708, and 2849. It can represent the entire sequencing result and meet the requirements of transcriptome data analysis.

### 3.7 Differentially expressed genes

**Figure 6:**
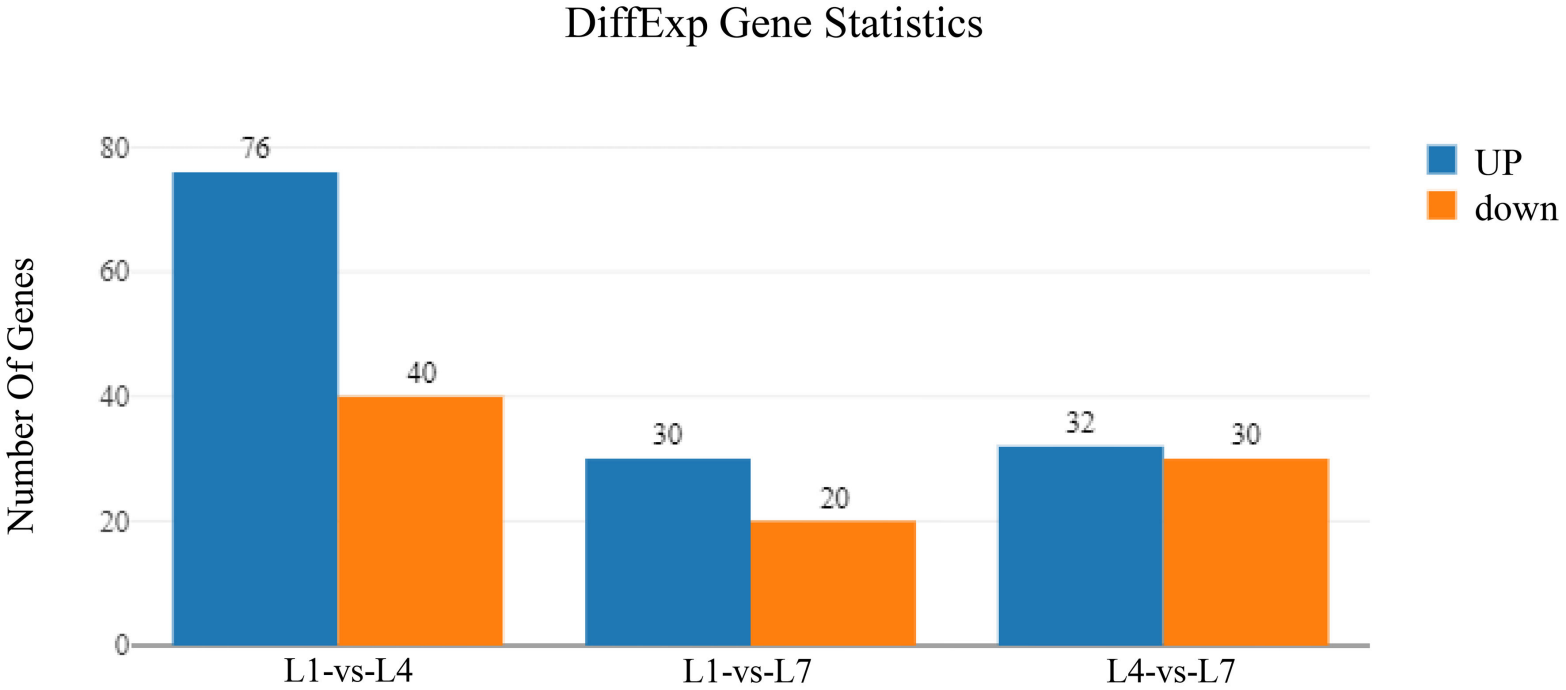
Differently-expressed genes of *Monopterus albus* liver in different levels of feed lipid.

By pairwise comparison of the differential genes in the three sets of libraries, the differential gene expression under different fat levels feeding conditions is shown in **Figure 6**. It was found that the L1-vs-L4, L1-vs-L7, and L4-vs-L7 groups had 116, 50, and 62 differentially expressed genes, respectively. The L1-vs-L4 group had 76 up-regulated genes and 40 down-regulated genes, the L1-vs-L7 group had 30 up-regulated genes and 20 down-regulated genes, and the L4-vs-L7 group had 32 up-regulated genes and 30 down-regulated genes.

### 3.8 Functional classification of differentially expressed genes by GO analysis

**Figure 7:**
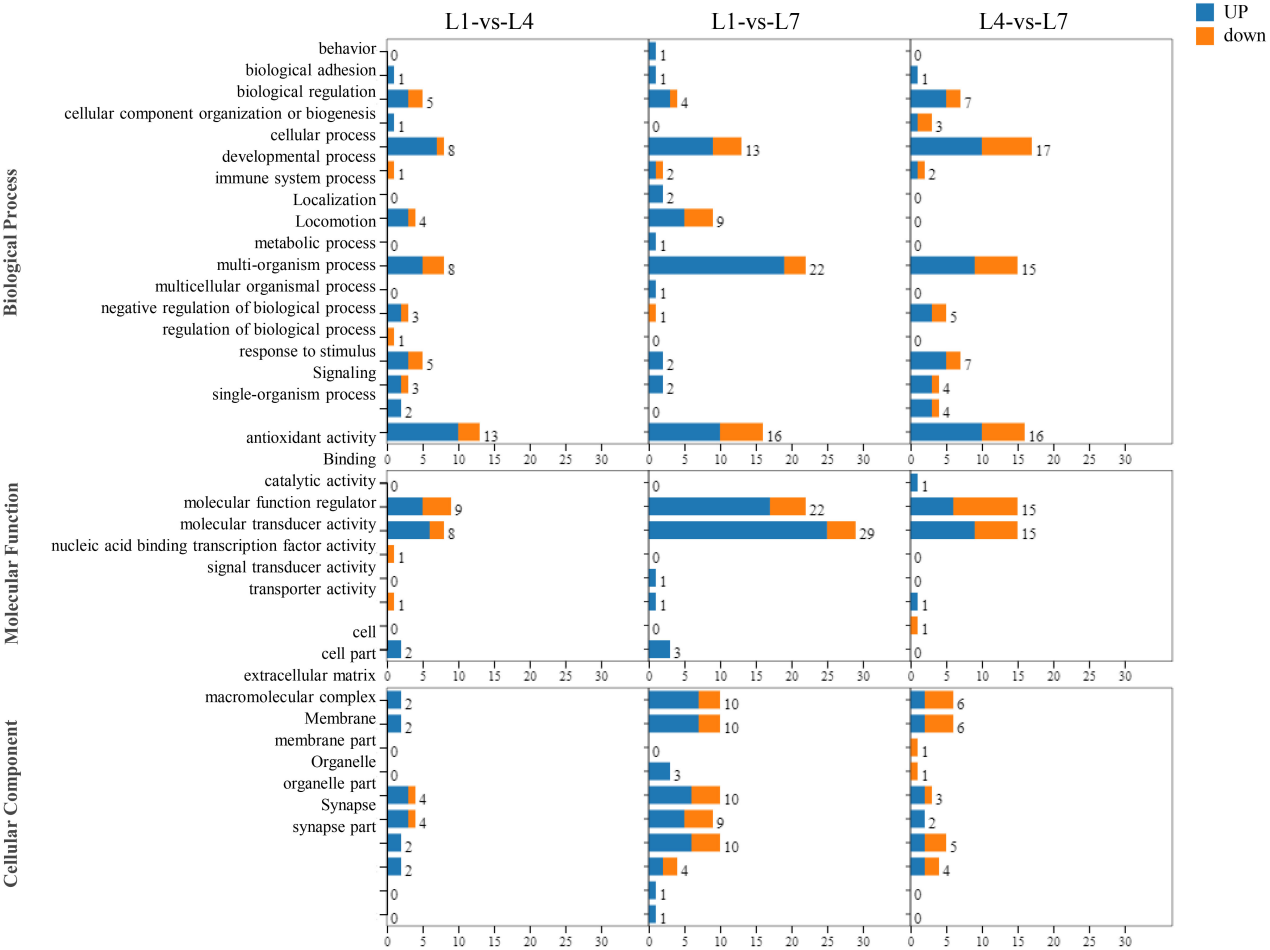
The function of the GO classification statistics of *Monopterus albus* liver in different levels of feed lipid.

As shown in **Figure 7**, by performing GO functional analysis of differential genes, the differentially expressed genes in the library can be classified into three categories: biological processes, molecular functions, and cellular components of 55 small categories. Genes related to cellular, metabolic, and single-organism processes were more enriched in the biological processes category. Among the molecular function processes category, binding and catalytically active processes are more enriched. More enriched genes were related to the cells and cell parts in the cellular component category. In addition, in L1-vs-L4 group, more differentially expressed genes were found than in the other groups.

### 3.9 KEGG pathway significant enrichment analysis

**Figure 8:**
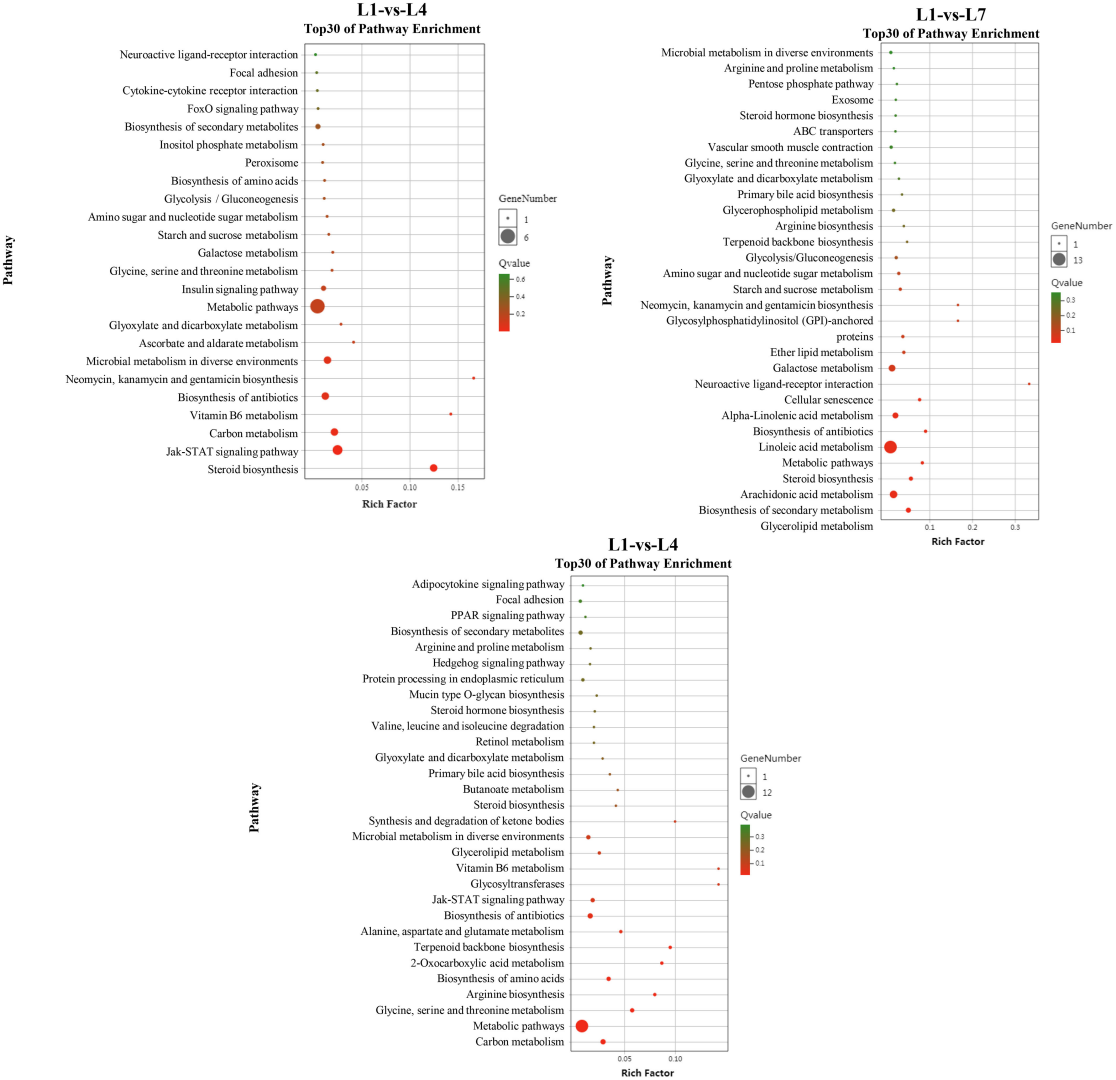
KEGG pathway enrichment analysis of *Monopterus albus* liver differentially expressed genes in different levels of feed lipid.

The differential genes between the three comparison groups were mapped to 226 specific metabolic pathways, of which 37 were significantly enriched metabolic pathways, to further study the relationship between the differentially expressed genes under different fat levels through KEGG enrichment analysis (**Figure 8**). Different fat levels affect the differential gene expression of swamp eel in glycerolipid metabolism (**Supplementary Figure 3**), glycolysis/gluconeogenesis (**Supplementary Figure 4**), ketone body synthesis, and degradation pathways (synthesis and degradation of ketone bodies, **Supplementary Figure 5**), the JAK-STAT signaling pathway (JAK-STAT signaling pathway, **Supplementary Figure 6**), and other pathways have significant enrichment.

### 3.10 Validation of the RNA-seq profiles by quantitative real-time PCR

**Figure 9:**
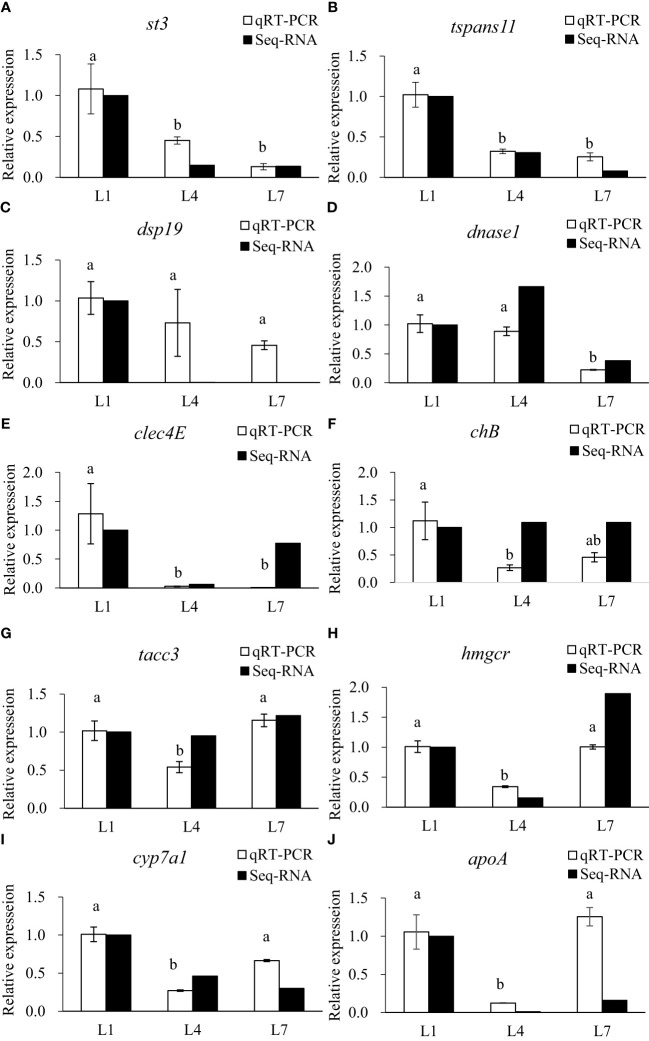
Validation of differential expression by quantitative real-time PCR. **(A)** stromelysin-3(st3); **(B)** tetraspanin-11(tspans11); **(C)** dual specificity protein phosphatase 19(dsp19); **(D)** deoxyribonuclease 1(dnase1); **(E)** C-type lectin domain family 4 member E(clec4E); **(F)** cornifelin homolog B(chB); **(G)** transforming acidic coiled-coil-containing protein 3(tacc3); **(H)** 3-hydroxy-3-methylglutaryl-coenzyme A reductase(hmgcr); **(I)** cholesterol 7-alpha-monooxygenase (cyp7a1); **(J)** apolipoprotein A(apoA). LF, MF and HF stand for feeding swamp eel with equal nitrogen feed with 0%, 6% and 12% lipid added to basic feed, respectively. Values of qRT-PCR are means ± SEM of three replicates, and values within the same row with different letters in superscript are significantly different (P < 0.05).

The qRT-PCR analysis of 10 randomly selected metabolic-related DEGs was performed to verify the reliability of the RNA-seq data. Select genes based on the functional enrichment and pathways of genes with different expression patterns. The expression trends were consistent for all genes, except for a difference in *chB*, as shown in **Figure 9**.

## 4 Discussion

Fish oil is generally believed to be the best lipid source for fish feed, and adding fish oil to feed can promote growth. Aquatic animals can effectively digest a certain amount of dietary lipids, and excess lipids will be lost with feces (31). Additionally, excessive dietary fat levels can impair the growth and survival of swamp eels, possibly due to reduced food intake caused by high-calorie content or the inability to metabolize high levels of lipids effectively. In this study, it was indicated by the results that too low and too high dietary lipid levels are not conducive to the growth of swamp eels. These results were similar to the studies in *Oreochromis niloticus×O. aureus (* 32), *Trichogaster trichopterus (* 33), and *Misgurnus anguillicaudatus (* 34). Based on our results, we estimate that the suitable feed lipid level of swamp eels is 7.03%.

Fish fat is generally deposited in the fat tissues around the abdominal viscera, head cavity, subcutaneous, and ventral side (35). The liver is usually considered the preferred storage organ for fat. Still, it has been shown in previous studies that the specific deposit location of fish fat varies by species. For instance, the fat of the *Atlantic halibut* is mainly deposited near bones and fins (36). However, *Ictalurus punctatus* fat is deposited in the abdominal cavity (37). Generally, whole-body lipid content is correlated with dietary lipid levels, and excessive dietary lipid results in excessive fat deposition in the liver, muscle, viscera, and adipose tissue of fishes (38). We also found equivalent results in our research regarding swamp eels. The fat content of the whole body, liver, intestine, muscle, and skin all increased with increased dietary lipid levels. We found in our research that the body fat of eels accumulates mainly in muscles at low dietary lipid levels. As dietary lipid levels increase, the fat content of eel skin is highest, followed by muscle. Its fat storage site is different from other fish (35). This is an indication that there may be a set of metabolic regulation pathways in the eel body that allow the avoidance of excessive fat deposition in the liver and intestines and affect their physiological functions. This has aroused great interest, and its regulatory mechanism needs further investigation.

Serum biochemical indices can reflect the nutrition status and health status when fish occurring pathological or physiological changes are affected by external factors (39). It was indicated in some reports that changes in fish serum biochemical indicators may be caused by stress or by some changes in nutrients in the diet (40). We showed in our results that high dietary fat levels significantly increase serum TG and TC, which agrees with previous reports in grass carp (38), largemouth bass (41), and turbot (42). GPT and GOT are generally used as indicators of cellular damage. The enzyme activity increases in the extracellular fluid and blood when cell damage occurs (43). An increase in plasma GPT and GOT activity is an indication of hepatic cell damage (43). In our study, it was found that elevated levels of dietary fat significantly increased the GPT and GOT activities in serum, suggesting that high-fat levels may cause some damage to the liver of the eel.

It was found in further research that high dietary lipid levels can cause excessive fat deposition in the liver, causing severe damage to hepatocytes. This is similar to a report on *Solea senegalensis* (44). This shows that high-fat feed causes excessive accumulation of fat in the liver of fish, thereby destroying liver metabolic function and tissue structure and seriously endangering fish health. However, the energy distribution of eels and the mechanism of fat deposition need to be further discussed.

RNA-seq is a new generation of high-throughput sequencing technology developed in recent years that has received widespread attention in studying the molecular mechanism of aquatic animals. For example, Tian et al. (17) analyzed the liver transcriptome of grass carp fed with fish oil and lard oil diets and revealed the importance of fish oil in feed at the molecular level. Deng et al. (45) also conducted an in-depth discussion of the molecular mechanism of the effects of feed ingredient substitution on the growth performance of Nile tilapia through transcriptomics. We performed liver transcriptome sequencing analysis of the 0% (L1), 6% (L4), and 12% (L7) groups to reveal further the molecular mechanism of the effects of different fat levels on the metabolic pathways of swamp eel. It was found that the L1-vs-L4, L1-vs-L7, and L4-vs-L7 groups had 116, 50, and 62 differentially expressed genes, respectively. These genes were compared to the KEGG database and enriched to multiple metabolic pathways. We are interested in the key pathways regulating glucolipid metabolism and energy balance in fish: the glycerolipid metabolism pathway, glycolysis/gluconeogenesis pathway, the synthesis and degradation of ketone bodies, and the JAK-STAT signaling pathway.

Adipose triacylglycerol lipase (ATGL; EC: 3.1.1.3) and the diacylglycerol acyltransferase (DGAT; EC: 2.3.1.20) in the glyceride metabolism pathway play critical roles in the decomposition and synthesis of triacylglycerol, respectively. In mammals, the first step of triglyceride hydrolysis has been shown to be selectively performed by ATGL, forming diglycerides and free fatty acids in adipocyte lipids (46). It has been increasingly demonstrated that the products and intermediates of lipolysis also have essential regulatory functions that affect cellular signals, gene expression, metabolism, cell growth, cell death, and lipotoxicity. Therefore, regulating *atgl* expression is critical to maintaining a balance between lipid storage and mobilization (47). The expression of *atgl* in mice can be upregulated by fasting and down-regulated by refeeding (46), and high-fat diets also reduce *atgl* expression in adipose tissue (48). Deiullis et al. (49) found that low-calorie intake can upregulate *atgl* gene expression in adult pigs. In yellow croakers, high-fat diets lead to a significant decrease in liver *atgl* expression (50). In this study, with the increase of lipid levels in feed, the crude fat content of the liver, intestine, muscle, skin, and whole fish in each experimental group increased. The expression of *atgl* in liver transcriptome also increased significantly. Although there is no research on the relationship between *atgl* expression and fat content in fish, we speculate that *atgl* transcription may play a significant role in regulating fish fat content.

Glycerolipids are obtained by modifying the glycerol 3-phosphate chain. Through the acylation and dephosphorylation reaction, glycerol 3-phosphate is converted to triacylglycerol. Regulation of the glycan-3-phosphate metabolic pathway is one of the methods used to control intracellular glycerolipid levels. Glycerol acyltransferases (GPATs), acylglycerol-phosphate acyltransferases (AGPATs), and diacylglycerol acyltransferases (DGATs) are essential enzymes driving the addition of fatty acids to the glycerol backbone (51). We screened *dgat* in the LF-vs-MF group for differentially expressed genes, which became another focus of our attention. It has been reported that using vegetable oil instead of fish oil in feed does not affect the DGAT activity of Atlantic salmon (52) and the expression of the *dgat* gene in European sea bass (53). Moreover, adding edible starch to the feed did not affect the expression of *dgat* in gilthead sea bream (54). In our study, we found that *dgat* was expressed significantly differently at distinct levels of fat feeding. We speculate that the glycerolipid synthesis process of swamp eel is regulated only by the lipid level in the diet and is not affected by the type of lipid or other nutrients. It may also be caused by different fat levels that change fatty acid levels and glycerol 3-phosphate levels in fish. A more in-depth regulation mechanism needs to be further explored.

After the animal eats, the carbohydrates in the food are digested by various glycosidases in the digestive tract. The monosaccharides produced are transported to different tissues to generate ATP through glycolysis to power the body (55). Excessive intake of carbohydrates can also cause glycogen or convert it to fat storage (56). Glycolysis is the key to energy production in most cells. The rate-limiting enzymes of this pathway include glucokinase (GK; EC: 2.7.1.2), which is strictly regulated by allosteric mediators and affects intracellular glucose metabolism (57). Meton et al. (58), found that feeding different levels of carbohydrates to feeds has essential effects on the critical enzymes in the glycolysis and glucose production processes of gilthead sea bream. Fernandez et al. (59) also found that a high proportion of carbohydrates in feed has an essential effect on the apparent digestibility, growth performance, and liver glucose metabolism pathways of gilthead sea bream. Lin et al. (60) reported in largemouth bass and *Micropterus salmoides* that the highest GK activity in the liver was observed in fish-fed high-starch diets. In our transcriptome data, the differentially expressed *gk* genes in the L1-vs-L4 and L1-vs-L7 groups were all enriched in the glycolysis pathway. The expression of GK in the L4 and L7 groups was significantly higher than in the L1 group. We hypothesize that different lipid levels affect the energy balance of the fish, further influencing the gluconeogenic pathway.

Differentially expressed genes in the L4 and L7 groups are enriched on multiple metabolic pathways. The synthesis and degradation of ketone bodies is a metabolic pathway that we pay special attention to. The ketone body is a fundamental energy supply substance that provides metabolic fuel for extrahepatic tissues with insufficient blood glucose (61). The production of ketone bodies occurs mainly in the mitochondria of liver cells. When blood glucose levels are low, mitochondria break down fatty acids to produce ketone bodies, which are then transported to various tissues to provide energy (62). In general, acetyl-CoA produced by the decomposition of fatty acids in the β-oxidation pathway enters the TCA cycle for oxidation (63).

Hydroxymethylglutaryl-CoA synthase (HMGCS; EC: 2.3.3.10) is a critical enzyme in synthesizing ketone bodies (64). In our results, the expression of *hmgcs* in the L7 group was much higher than that in the L4 group, suggesting that the rate of ketone body synthesis in the L7 group was higher than in the L4 group. This may be due to the elevated level of lipid intake that accelerates the β-oxidation of fatty acids in swamp eel, resulting in excess acetyl-CoA, which activates the ketone body production process. When the body has sufficient energy, the ketone bodies produced cannot be effectively used, and excessive ketone concentrations harm the body. This may also be one of the reasons why excessive lipid levels are not conducive to the growth of swamp eel.

The JAK-STAT signaling pathway is a pleiotropic cascade that plays an essential role in the intracellular signals of lipid metabolism (65). It has been reported that the disruption of the JAK/STAT signaling pathway can lead to dysregulation of hepatic glucose production, hepatic steatosis, and insulin resistance (66). Lack of STAT3 in hepatocytes can also lead to increased insulin resistance and increased expression of gluconeogenesis-related genes (67). Targeted deletion of JAK2 (68) or STAT5 (69) in the liver can also cause severe fat accumulation and hepatic steatosis. The L1-vs-L7 and L4-vs-L7 groups are enriched with various genes in the JAK-STAT signaling pathway, indicating that high lipid levels can regulate glucolipid metabolism and energy balance in swamp eel through the JAK-STAT signaling pathway.

## 5 Conclusion

In this report, the appropriate lipid level (7.03%) in feed can promote the growth of swamp eel and maintain active glucolipid metabolism. However, excessively high lipid levels can cause increased blood lipids and disturbances in glucolipid metabolism, resulting in hepatocyte damage and hepatic steatosis. Further research through high-throughput sequencing technology found that the regulatory mechanism may involve the glycerolipid metabolism pathway, glycolysis/gluconeogenesis pathway, synthesis and degradation of ketone bodies, and JAK-STAT signaling pathway.

## Data availability statement

The original contributions presented in the study are publicly available. This data can be found here:10.6084/m9.figshare.23465612.

## Ethics statement

The animal study was reviewed and approved by China Law for Animal Health Protection and Instructions.

## Author contributions

YZ, ZW, and QZ contributed to conception and design of the study. FG, XY and YL organized the database. ZH performed the statistical analysis. YZ wrote the first draft of the manuscript. YB, ZW, and QZ wrote sections of the manuscript. All authors contributed to the article and approved the submitted version.

## Supplementary material

Supplementary Figure 1 Analysis of transcriptomics sequence saturation of *Monopterus albus* liver under different levels of feed lipid.

Supplementary Figure 2 Gene coverage statistics of transcriptomics sequencing of *Monopterus albus* liver in different levels of feed lipid.

Supplementary Figure 3 Differentially expressed genes location in pathway of glycerolipid metabolism.

Supplementary Figure 4 Differentially expressed genes location in pathway of glycplysis/gluconeogensis.

Supplementary Figure 5 Differentially expressed genes location in pathway of synthesis and degradation of ketone bodies.

Supplementary Figure 6 Differentially expressed genes location in pathway of JAK-STAT signaling pathway.

## References

[1] Polakof S Moon TW Aguirre P Skiba-Cassy S Panserat S. Effects of insulin infusion on glucose homeostasis and glucose metabolism in rainbow trout fed a high-carbohydrate diet. J Exp Biol (2010) 213(Pt 24):4151–7. doi: 10.1242/jeb.050807 21112995

[2] Tocher DR. Metabolism and functions of lipids and fatty acids in teleost fish. Rev Fish. Sci (2003) 11(2):107–84. doi: 10.1080/713610925

[3] Yuan D Zhou C Wang T Lin F Chen H Wu H. Molecular characterization and tissue expression of peptide YY in schizothorax prenanti: effects of periprandial changes and fasting on expression in the hypothalamus. Regul Peptides (2014) 190-191:32–8. doi: 10.1016/j.regpep.2014.03.004 24681121

[4] Helland SJ Grisdale-Helland B. The influence of replacing fish meal in the diet with fish oil on growth, feed utilization and body composition of Atlantic salmon (*Salmo salar*) during the smoltification period. Aquaculture (1998) 162(1-2):1–10. doi: 10.1016/S0044-8486(98)00206-3

[5] Ma Q Li L-Y Le J-Y Lu D-L Qiao F Zhang M-L. Dietary microencapsulated oil improves immune function and intestinal health in Nile tilapia fed with high-fat diet. Aquaculture (2018) 496:19–29. doi: 10.1016/j.aquaculture.2018.06.080

[6] Tang T Hu Y Peng M Chu W Hu Y Zhong L. Effects of high-fat diet on growth performance, lipid accumulation and lipid metabolism-related MicroRNA/gene expression in the liver of grass carp (*Ctenopharyngodon idella*). Comp Biochem Physiol Part B Biochem Mol Biol (2019) 234:34–40. doi: 10.1016/j.cbpb.2019.04.006 31071453

[7] Naiel MAE Negm SS Ghazanfar S Shukry M. The risk assessment of high-fat diet in farmed fish and its mitigation approaches: a review. J Anim Physiol Anim Nutr (Berl) (2023) 107(3):948–69. doi: 10.1111/jpn.13759 35934925

[8] Chen Y Shen Y Pandit NP Fu J Li D Li J. Molecular cloning, expression analysis, and potential food intake attenuation effect of peptide YY in grass carp (*Ctenopharyngodon idellus*). Gen Comp Endocrinol (2013) 187:66–73. doi: 10.1016/j.ygcen.2013.03.029 23583472

[9] Yan P Jia J Yang G Wang D Sun C Li W. Duplication of neuropeptide y and peptide YY in Nile tilapia oreochromis niloticus and their roles in food intake regulation. Peptides (2017) 88:97–105. doi: 10.1016/j.peptides.2016.12.010 27988351

[10] Cao XF Dai YJ Liu MY Yuan XY Wang CC Huang YY. High-fat diet induces aberrant hepatic lipid secretion in blunt snout bream by activating endoplasmic reticulum stress-associated IRE1/XBP1 pathway. Biochim Biophys Acta Mol Cell Biol Lipids (2019) 1864(3):213–23. doi: 10.1016/j.bbalip.2018.12.005 30553054

[11] Jin M Pan T Tocher DR Betancor MB Monroig Ó Shen Y. Dietary choline supplementation attenuated high-fat diet-induced inflammation through regulation of lipid metabolism and suppression of NFκB activation in juvenile black seabream (*Acanthopagrus schlegelii*). J Nutr Sci (2019) 8:e38. doi: 10.1017/jns.2019.34 32042405 PMC6984006

[12] Liu G Yu H Wang C Li P Liu S Zhang X. Nano−selenium supplements in high-fat diets relieve hepatopancreas injury and improve survival of grass carp ctenopharyngodon idella by reducing lipid deposition. Aquaculture (2021) 538:736580. doi: 10.1016/j.aquaculture.2021.736580

[13] Li A Yuan X Liang X-F Liu L Li J Li B. Adaptations of lipid metabolism and food intake in response to low and high fat diets in juvenile grass carp (*Ctenopharyngodon idellus*). Aquaculture (2016) 457:43–9. doi: 10.1016/j.aquaculture.2016.01.014

[14] Jia R Cao LP Du JL He Q Gu ZY Jeney G. Effects of high-fat diet on antioxidative status, apoptosis and inflammation in liver of tilapia (*Oreochromis niloticus*) *via* Nrf2, TLRs and JNK pathways. Fish Shellfish Immunol (2020) 104:391–401. doi: 10.1016/j.fsi.2020.06.025 32553566

[15] Gonzalez R Unniappan S. Molecular characterization, appetite regulatory effects and feeding related changes of peptide YY in goldfish. Gen Comp Endocrinol (2010) 166(2):273–9. doi: 10.1016/j.ygcen.2009.09.008 19800344

[16] Liu Z-L Zhao W Hu W-S Zhu B Xie J-J Liu Y-J. Lipid metabolism, growth performance, antioxidant ability and intestinal morphology of rainbow trout (*Oncorhynchus mykiss*) under cage culture with flowing water were affected by dietary lipid levels. Aquac. Rep (2021) 19:100593. doi: 10.1016/j.aqrep.2021.100593

[17] Tian JJ Lu RH Ji H Sun J Li C Liu P. Comparative analysis of the hepatopancreas transcriptome of grass carp (*Ctenopharyngodon idellus*) fed with lard oil and fish oil diets. Gene (2015) 565(2):192–200. doi: 10.1016/j.gene.2015.04.010 25865300

[18] Bottcher G Ahren B Lundquist I Sundler F. Peptide YY: intrapancreatic localization and effects on insulin and glucagon secretion in the mouse. Pancreas (1989) 4(3):282–8. doi: 10.1097/00006676-198906000-00002 2660131

[19] Castan I Valet P Larrouy D Voisin T Remaury A Daviaud D. Distribution of PYY receptors in human fat cells: an antilipolytic system alongside the alpha 2-adrenergic system. Am J Physiol (1993) 265(1 Pt 1):E74–80. doi: 10.1152/ajpendo.1993.265.1.E74 8393293

[20] Boey D Lin S Karl T Baldock P Lee N Enriquez R. Peptide YY ablation in mice leads to the development of hyperinsulinaemia and obesity. Diabetologia (2006) 49(6):1360–70. doi: 10.1007/s00125-006-0237-0 16680491

[21] Salmerón C. Adipogenesis in fish. J Exp Biol (2018) 221(Pt Suppl 1):jeb161588. doi: 10.1242/jeb.161588 29514876

[22] Marques VH Moreira RG Branco GS Honji RM Rombenso AN Viana MT. Different saturated and monounsaturated fatty acids levels in fish oil-free diets to cobia (*Rachycentron canadum*) juveniles: effects in growth performance and lipid metabolism. Aquaculture (2021) 541:736843. doi: 10.1016/j.aquaculture.2021.736843

[23] Araújo BC Rodriguez M Honji RM Rombenso AN del Rio-Zaragoza OB Cano A. Arachidonic acid modulated lipid metabolism and improved productive performance of striped bass (*Morone saxatilis*) juvenile under sub-to optimal temperatures. Aquaculture (2021) 530:735939. doi: 10.1016/j.aquaculture.2020.735939

[24] Kim D Langmead B Salzberg SL. HISAT: a fast spliced aligner with low memory requirements. Nat Methods (2015) 12(4):357–60. doi: 10.1038/nmeth.3317 PMC4655817 25751142

[25] Robinson MD Oshlack A. A scaling normalization method for differential expression analysis of RNA-seq data. Genome Biol (2010) 11(3):R25. doi: 10.1186/gb-2010-11-3-r25 20196867 PMC2864565

[26] Bauer S Grossmann S Vingron M Robinson PN. Ontologizer 2.0–a multifunctional tool for GO term enrichment analysis and data exploration. Bioinf (Oxford England) (2008) 24(14):1650–1. doi: 10.1093/bioinformatics/btn250 18511468

[27] Ye J Fang L Zheng H Zhang Y Chen J Zhang Z. WEGO: a web tool for plotting GO annotations. Nucleic Acids Res (2006) 34(Web Server issue):W293–7. doi: 10.1093/nar/gkl031 PMC1538768 16845012

[28] Kanehisa M Goto S Furumichi M Tanabe M Hirakawa M. KEGG for representation and analysis of molecular networks involving diseases and drugs. Nucleic Acids Res (2010) 38(suppl_1):D355–D60. doi: 10.1093/nar/gkp896 PMC2808910 19880382

[29] Zhang Y Tang Z Lin W Yuan X Jia J Sun C. Molecular identification, tissue distribution and functional analysis of somatostatin receptors (SSTRs) in red-spotted grouper (*Epinephelus akaara*). Gen Comp Endocrinol (2019) 274:87–96. doi: 10.1016/j.ygcen.2019.01.007 30654020

[30] Schmittgen TD Livak KJ. Analyzing real-time PCR data by the comparative C(T) method. Nat Protoc (2008) 3(6):1101–8. doi: 10.1038/nprot.2008.73 18546601

[31] Izquierdo MS Socorro J Arantzamendi L Hernández-Cruz CM. Recent advances in lipid nutrition in fish larvae. Fish Physiol Biochem (2000) 22(2):97–107. doi: 10.1023/A:1007810506259

[32] Han CY Wen XB Zheng QM Li HB. Effects of dietary lipid levels on lipid deposition and activities of lipid metabolic enzymes in hybrid tilapia (Oreochromis niloticus × o. aureus). J Anim Physiol Anim Nutr (Berl) (2011) 95(5):609–15. doi: 10.1111/j.1439-0396.2010.01091.x 21114544

[33] Mohanta KN Subramanian S Korikanthimath VS. Effect of dietary protein and lipid levels on growth, nutrient utilization and whole-body composition of blue gourami, trichogaster trichopterus fingerlings. J Anim Physiol Anim Nutr (Berl) (2013) 97(1):126–36. doi: 10.1111/j.1439-0396.2011.01258.x 22129348

[34] Li Y Jia Z Liang X Matulic D Hussein M Gao J. Growth performance, fatty-acid composition, lipid deposition and hepatic-lipid metabolism-related gene expression in juvenile pond loach misgurnus anguillicaudatus fed diets with different dietary soybean oil levels. J Fish Biol (2018) 92(1):17–33. doi: 10.1111/jfb.13472 29148037

[35] Toussaint C Fauconneau B Médale F Collewet G Akoka S Haffray P. Description of the heterogeneity of lipid distribution in the flesh of brown trout ( *Salmo trutta* ) by MR imaging. Aquaculture (2005) 243(1):255–67. doi: 10.1016/j.aquaculture.2004.09.029

[36] Berge GM Storebakken T. Effect of dietary fat level on weight gain, digestibility, and fillet composition of Atlantic halibut. Aquaculture (1991) 99(3-4):331–8. doi: 10.1016/0044-8486(91)90253-4

[37] Gaylord TG Gatlin DM III. Dietary protein and energy modifications to maximize compensatory growth of channel catfish (*Ictalurus punctatus*). Aquaculture (2001) 194(3-4):0–348. doi: 10.1016/S0044-8486(00)00523-8

[38] Yuan X Liang XF Liu L Fang J Li J Li A. Fat deposition pattern and mechanism in response to dietary lipid levels in grass carp, ctenopharyngodon idellus. Fish Physiol Biochem (2016) 42(6):1557–69. doi: 10.1007/s10695-016-0240-4 27216495

[39] Shi G. Effects of dietary lipid level on growth performance of genetic improvement of farmed Tilapia(GIFT,*Oreochromis niloticus*) and its serum biochemical indices and fatty acid composition under cold stress. Chin J Anim Nutr (2012) 24(11):2154–64. doi: 10.1007/s11783-011-0280-z

[40] Lin H Tan X Zhou C Niu J Xia D Huang Z. Effect of dietary arginine levels on the growth performance, feed utilization, non-specific immune response and disease resistance of juvenile golden pompano trachinotus ovatus. Aquaculture (2015) 437:382–9. doi: 10.1016/j.aquaculture.2014.12.025

[41] Zhou YL Guo JL Tang RJ Ma HJ Chen YJ Lin SM. High dietary lipid level alters the growth, hepatic metabolism enzyme, and anti-oxidative capacity in juvenile largemouth bass micropterus salmoides. Fish Physiol Biochem (2020) 46(1):125–34. doi: 10.1007/s10695-019-00705-7 31522360

[42] Christelle R Jacqueline A Mireille C Jean R Sadasivam K. Dietary lipid level, hepatic lipogenesis and flesh quality in turbot (*Psetta maxima*). Aquaculture (2001) 193(3):291–309. doi: 10.1016/S0044-8486(00)00493-2

[43] Ramesh M Sankaran M Veera-Gowtham V Poopal RK. Hematological, biochemical and enzymological responses in an Indian major carp labeo rohita induced by sublethal concentration of waterborne selenite exposure. Chemico Biol Interact (2014) 207:67–73. doi: 10.1016/j.cbi.2013.10.018 24183823

[44] Mandrioli L Sirri R Gatta PP Morandi F Sarli G Parma L. Histomorphologic hepatic features and growth performances of juvenile Senegalese sole (*Solea senegalensis*) fed isogenertic practical diets with variable protein/lipid levels. J Appl Ichthyology (2012) 28(4):0–. doi: 10.1111/j.1439-0426.2012.01938.x

[45] Deng J Mai K Chen L Mi H Zhang L. Effects of replacing soybean meal with rubber seed meal on growth, antioxidant capacity, non-specific immune response, and resistance to aeromonas hydrophila in tilapia (Oreochromis niloticus x O. aureus). Fish Shellfish Immunol (2015) 44(2):436–44. doi: 10.1016/j.fsi.2015.03.018 25804486

[46] Kershaw EE Hamm JK Verhagen LA Peroni O Katic M Flier JS. Adipose triglyceride lipase: function, regulation by insulin, and comparison with adiponutrin. Diabetes (2006) 55(1):148–57. doi: 10.2337/diabetes.55.01.06.db05-0982 PMC2819178 16380488

[47] Cerk IK Wechselberger L Oberer M. Adipose triglyceride lipase regulation: an overview. Curr Protein Pept Sci (2018) 19(2):221–33. doi: 10.2174/1389203718666170918160110 PMC7613786 28925902

[48] Shen WJ Patel S Yu Z Jue D Kraemer FB. Effects of rosiglitazone and high fat diet on lipase/esterase expression in adipose tissue. Biochim Biophys Acta (2007) 1771(2):177–84. doi: 10.1016/j.bbalip.2006.11.009 PMC1933526 17215164

[49] Deiuliis JA Shin J Bae D Azain MJ Barb R Lee K. Developmental, hormonal, and nutritional regulation of porcine adipose triglyceride lipase (ATGL). Lipids (2008) 43(3):215–25. doi: 10.1007/s11745-007-3146-1 18189154

[50] Wang X Wang Y Li Y. Adipose triglyceride lipase (ATGL) clone, expression pattern, and regulation by different lipid sources and lipid levels in large yellow croaker (Pseudosciaena crocea r.). Mar Biotechnol (New York NY) (2013) 15(2):197–205. doi: 10.1007/s10126-012-9477-9 22836232

[51] Zhang P Reue K. Lipin proteins and glycerolipid metabolism: roles at the ER membrane and beyond. Biochim Biophys Acta Biomembr (2017) 1859(9 Pt B):1583–95. doi: 10.1016/j.bbamem.2017.04.007 PMC5688847 28411173

[52] Oxley A Torstensen BE Rustan AC Olsen RE. Enzyme activities of intestinal triacylglycerol and phosphatidylcholine biosynthesis in Atlantic salmon (Salmo salar l. Comp Biochem Physiol Part B Biochem Mol Biol (2005) 141(1):77–87. doi: 10.1016/j.cbpc.2005.01.012 15820137

[53] Castro CO Corraze G Panserat S Oliva-Teles A. Effects of fish oil replacement by a vegetable oil blend on digestibility, postprandial serum metabolite profile, lipid and glucose metabolism of European sea bass (*Dicentrarchus labrax*) juveniles. Aquac. Nutr (2015) 21(5):592–603. doi: 10.1111/anu.12184

[54] Castro C Corraze G Basto A Larroquet L Panserat S Oliva-Teles A. Dietary lipid and carbohydrate interactions: implications on lipid and glucose absorption, transport in gilthead Sea bream (*Sparus aurata*) juveniles. Lipids (2016) 51(6):743–55. doi: 10.1007/s11745-016-4140-2 27023202

[55] Nordlie RC Foster JD Lange AJ. Regulation of glucose production by the liver. Annu Rev Nutr (1999) 19:379–406. doi: 10.1146/annurev.nutr.19.1.379 10448530

[56] Towle HC Kaytor EN Shih HM. Regulation of the expression of lipogenic enzyme genes by carbohydrate. Annu Rev Nutr (1997) 17:405–33. doi: 10.1146/annurev.nutr.17.1.405 9240934

[57] Pilkis SJ Claus TH. Hepatic gluconeogenesis/glycolysis: regulation and structure/function relationships of substrate cycle enzymes. Annu Rev Nutr (1991) 11:465–515. doi: 10.1146/annurev.nu.11.070191.002341 1892710

[58] Meton I Mediavilla D Caseras A Canto E Fernandez F Baanante IV. Effect of diet composition and ration size on key enzyme activities of glycolysis-gluconeogenesis, the pentose phosphate pathway and amino acid metabolism in liver of gilthead sea bream (Sparus aurata). Br J Nutr (1999) 82(3):223–32. doi: 10.1017/S0007114599001403 10655969

[59] Fernández F Miquel AG Córdoba M Varas M Metón I Caseras A. Effects of diets with distinct protein-to-carbohydrate ratios on nutrient digestibility, growth performance, body composition and liver intermediary enzyme activities in gilthead sea bream (Sparus aurata, l.) fingerlings. J Exp Mar Biol Ecol (2007) 343(1):1–10. doi: 10.1016/j.jembe.2006.10.057

[60] Lin S-M Shi C-M Mu M-M Chen Y-J Luo L. Effect of high dietary starch levels on growth, hepatic glucose metabolism, oxidative status and immune response of juvenile largemouth bass, micropterus salmoides. Fish Shellfish Immunol (2018) 78:121–6. doi: 10.1016/j.fsi.2018.04.046 29684600

[61] Morris AA. Cerebral ketone body metabolism. J Inherited Metab Dis (2005) 28(2):109–21. doi: 10.1007/s10545-005-5518-0 15877199

[62] McPherson PA McEneny J. The biochemistry of ketogenesis and its role in weight management, neurological disease and oxidative stress. J Physiol Biochem (2012) 68(1):141–51. doi: 10.1007/s13105-011-0112-4 21983804

[63] Rui L. Energy metabolism in the liver. Compr Physiol (2014) 4(1):177–97. doi: 10.1002/cphy.c130024 PMC4050641 24692138

[64] Kang HB Fan J Lin R Elf S Ji Q Zhao L. Metabolic rewiring by oncogenic BRAF V600E links ketogenesis pathway to BRAF-MEK1 signaling. Mol Cell (2015) 59(3):345–58. doi: 10.1016/j.molcel.2015.05.037 PMC4530073 26145173

[65] Richard AJ Stephens JM. The role of JAK-STAT signaling in adipose tissue function. Biochim Biophys Acta (2014) 1842(3):431–9. doi: 10.1016/j.bbadis.2013.05.030 PMC4029773 23735217

[66] Dodington DW Desai HR Woo M. JAK/STAT - emerging players in metabolism. Trends Endocrinol Metab: TEM (2018) 29(1):55–65. doi: 10.1016/j.tem.2017.11.001 29191719

[67] Ramadoss P Unger-Smith NE Lam FS Hollenberg AN. STAT3 targets the regulatory regions of gluconeogenic genes in vivo. Mol Endocrinol (Baltimore Md) (2009) 23(6):827–37. doi: 10.1210/me.2008-0264 PMC5419286 19264844

[68] Shi SY Martin RG Duncan RE Choi D Lu SY Schroer SA. Hepatocyte-specific deletion of janus kinase 2 (JAK2) protects against diet-induced steatohepatitis and glucose intolerance. J Biol Chem (2012) 287(13):10277–88. doi: 10.1074/jbc.M111.317453 PMC3323042 22275361

[69] Hosui A Tatsumi T Hikita H. Signal transducer and activator of transcription 5 plays a crucial role in hepatic lipid metabolism through regulation of CD36 expression. Hepatol Res(2017) 47(8):813–25. doi: 10.1111/hepr.12816 27593674

