# Supplementary figures and images for: Insights into the mechanism of growth and fat deposition by feeding different levels of lipid provided by transcriptome analysis of swamp eel (Monopterus albus, Zuiew 1793) liver
Source: Front Immunol. 2023 Jun 19;14:1118198. doi: 10.3389/fimmu.2023.1118198 (PMC10315655; doi:10.3389/fimmu.2023.1118198)

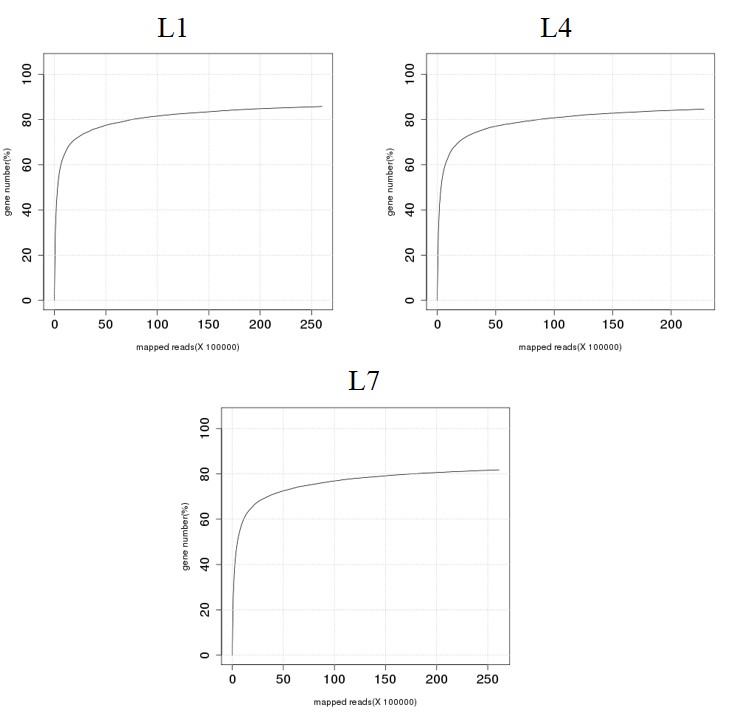

Supplement: Supplementary Figure 1 — Analysis of transcriptomics sequence saturation of Monopterus albus liver under different levels of feed lipid. [file Image_1.jpeg]

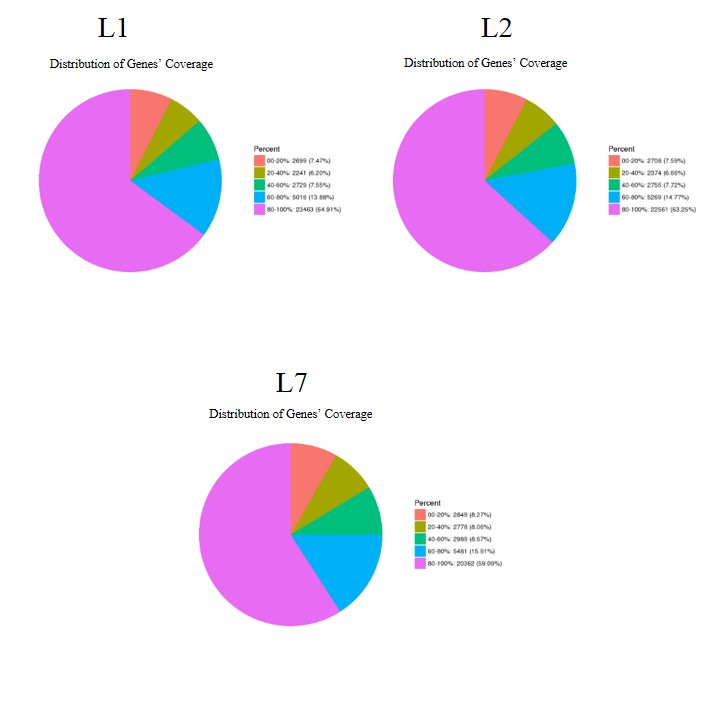

Supplement: Supplementary Figure 2 — Gene coverage statistics of transcriptomics sequencing of Monopterus albus liver in different levels of feed lipid. [file Image_2.jpeg]

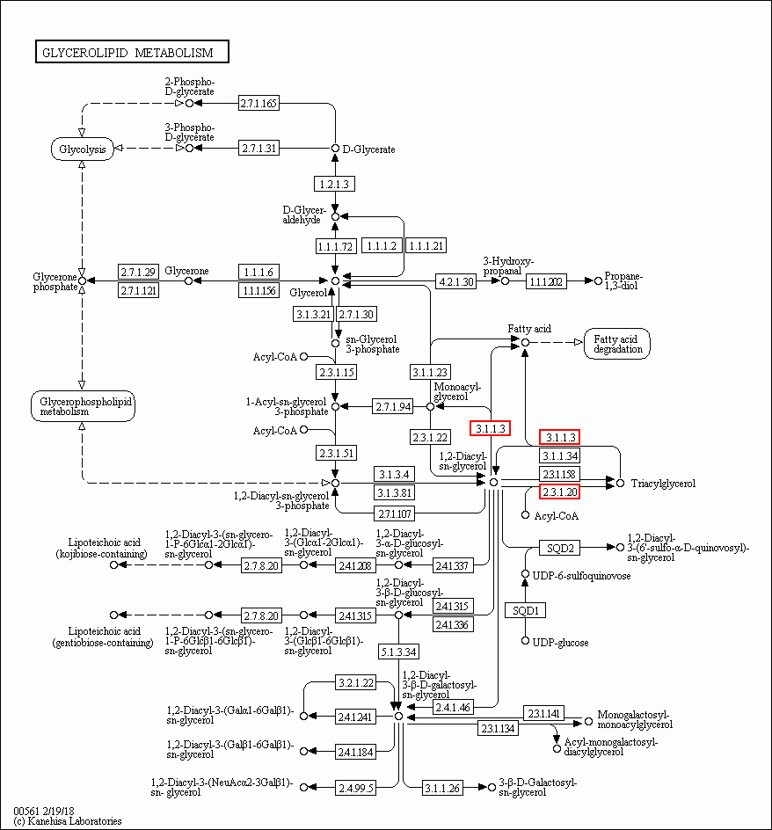

Supplement: Supplementary Figure 3 — Differentially expressed genes location in pathway of glycerolipid metabolism. [file Image_3.jpeg]

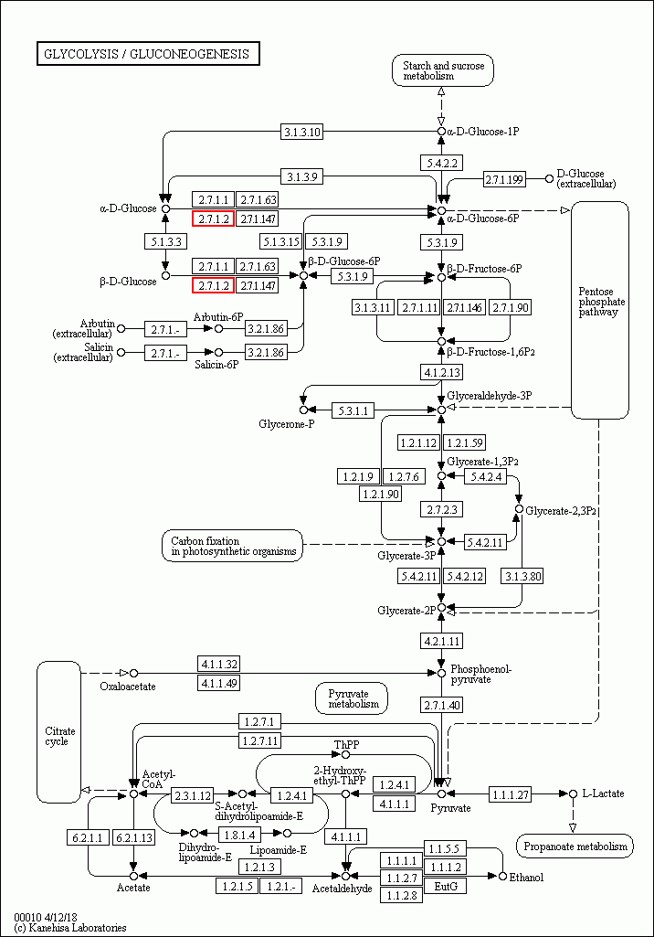

Supplement: Supplementary Figure 4 — Differentially expressed genes location in pathway of glycplysis/gluconeogensis. [file Image_4.jpeg]

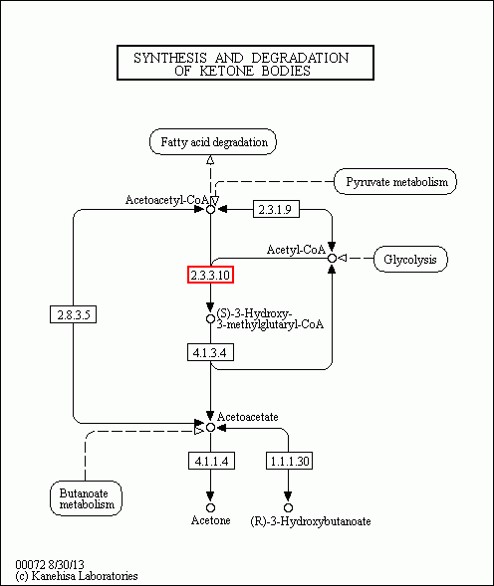

Supplement: Supplementary Figure 5 — Differentially expressed genes location in pathway of synthesis and degradation of ketone bodies. [file Image_5.jpeg]

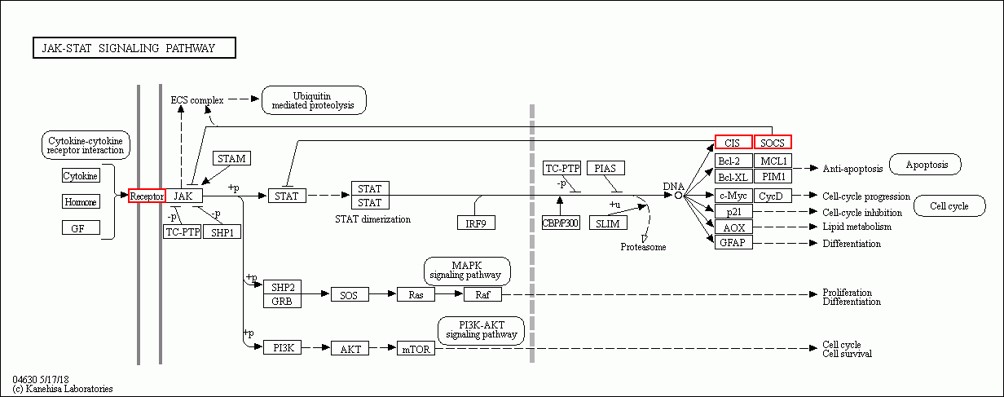

Supplement: Supplementary Figure 6 — Differentially expressed genes location in pathway of JAK-STAT signaling pathway. [file Image_6.jpeg]
