# Supplementary material for: Insights into the mechanism of growth and fat deposition by feeding different levels of lipid provided by transcriptome analysis of swamp eel (Monopterus albus, Zuiew 1793) liver
Source: Front Immunol. 2023 Jun 19;14:1118198. doi: 10.3389/fimmu.2023.1118198 (PMC10315655; doi:10.3389/fimmu.2023.1118198)
Supplement: Supplementary file 7 [file Table_1.docx]

**Supplementary Table 1** Information of primer used in real-time PCR

| Name | Primer sequence (5’-3’) | Genbank accession no. |
| --- | --- | --- |
| *β-actin* F | CTGGACTTCGAGCAGGAGAT | AY647143 |
| *β-actin* R | ACCAAGGAAAGAAGGCTGGA |  |
| *st3* F | CCAGCCAACCCCGAGACCAAT | XM_020595316.1 |
| *st3* R | TCTTCAAAGGCGGCGTCAATA |  |
| *tspans11* F | CTTGAGTCTGCTGGCGTCC | XM_020599615.1 |
| *tspans11* R | TCCCTGGCTGGGCATAGTT |  |
| *dsp19* F | CTCGGACACGGCTGAGGAAG | XM_020623843.1 |
| *dsp19* R | GATGAACAGGTCGGGAAACA |  |
| *dnase1* F | AGCCTCTGGGTCTGAACTCCTA | XM_020613243.1 |
| *dnase1* R | ACTTCCTCCACTGCCTTGTCTG |  |
| *tacc3* F  *tacc3* R | ATTTGGCACAACAACAAGCATA  TTGAGTTCAGTTACTGGGACAT | XM_020603340.1 |
| *hmgcr* F | ATGCTGTTGGTCAATTCTAATC | XM_020599204.1 |
| *hmgcr* R | AAATACTTTCCATCCAGGTGC |  |
| *clec4E* F | AATCAACAAGGGAGGAAAAC | XM_020590416.1 |
| *clec4E* R | TCCACAAGTCCAATCCAGTA |  |
| *chB* F | GACGATGTGCCTGGATGCTGTT | XM_020625583.1 |
| *chB* R | GGTGCCTTTGATGCCATAACGA |  |
| *cyp7a1* F | ACGCTGTTTGGTAAGGAGTT | XM_020613358.1 |
| *cyp7a1* R | TCTGGCACTGTAGGCACTCT |  |
| *apoA* F | AGGCTTACGACAAGACAGAACT | XM_020600117.1 |
| *apoA* R | CGAAGACGACTACGGAGACGAT |  |
